# Analysis of female pre-clinical students’ readiness, academic performance and satisfaction in online learning: an assessment of quality for curriculum revision and future implementation

**DOI:** 10.1186/s12909-023-04503-x

**Published:** 2023-07-21

**Authors:** Kavitha Ganesh, Najwa Abdur Rashid, Raja El Hasnaoui, Rasha Assiri, Mary Anne W. Cordero

**Affiliations:** College of Medicine, Princess Nourah Bint Abdulrahman University, P.O. Box 84428, Riyadh, 11671 Saudi Arabia

**Keywords:** Online learning, Face-to-face learning, Online learning readiness, Practical skills, Academic performance

## Abstract

**Background:**

The acceptance of online courses by medical and dental students, especially during the coronavirus disease 2019 crisis, is substantial, as reported in various studies. However, the unfavourable online learning experiences of the students during the pandemic were also highlighted. As the teaching-learning process is returning to the “new normal,“ it is necessary to identify online learning domains implemented during the pandemic crisis that may be applied in pre-clinical courses in the future.

**Methods:**

A validated Student Online Learning Readiness questionnaire assessed pre-clinical students’ online learning competence. Students’ academic performance in face-to-face post-pandemic was compared with their performance in online settings during the pandemic crisis. Students’ satisfaction with online learning was evaluated using a self-made survey questionnaire. Descriptive statistics, the t-test, and multiple regression analysis were used to analyze the data gathered with a p-value ≤ 0.05 considered statistically significant.

**Results:**

Except for social skills with classmates and groupmates, in which 47.5% of respondents indicated unreadiness, most students were prepared for online learning. Theory-wise, online learners outperformed traditional learners, but the difference was insignificant. In contrast, students’ practical skills in face-to-face modality are significantly higher (p = 0.029). Students rated their satisfaction with online learning higher for interactions with instructors and staff and lower for interactions with classmates and group mates and skill acquisition.

**Conclusion:**

Providing high-quality pre-clinical online teaching was achieved for theoretical components but not practical skills acquisition. Students’ social engagement with peers is one of the key elements crucial to online learning success. Academic leaders and curriculum developers must recognize potential gaps as they transition to online learning.

## Introduction

Medical education has transitioned from traditional teaching techniques to other media that involve online or electronic learning [1]. The acceptance of evidence-based teaching practices has accompanied this transformation and the widespread usage of novel learning approaches supported by digital technology [2–4]. According to several studies, technology-based medical curricula are more effective than conventional methods and are highly regarded by medical organizations [2, 4–7]. One of the cutting-edge teaching techniques that today’s students are interested in is online learning (OL), a method of education that caters to creative students in remote regions who are unable to attend face-to-face classes [8]. OL has gained popularity as a teaching technique because it enables the acquisition of knowledge using various media in an OL environment [9]. Globally, educational institutions were compelled to transition from traditional face-to-face to OL in response to the COVID-19 pandemic.

OL offers opportunities and challenges, enabling a learning process not confined to the classroom walls [10]. Access to information regardless of location, a more personalized learning experience, cost-effectiveness, a wider breadth of learning depending on individual interests, and flexibility in sharing knowledge with others are only a few of the critical advantages of the OL modality [9, 11]. However, other studies have shown that OL is inferior to face-to-face learning since students need more opportunities to socialize and develop their interpersonal skills and limited interaction and discussion between students and teachers [12–15]. Others are concerned that the increased likelihood of feeling lost, alone, and disappointed among online learners may limit or reduce their capacity to learn successfully and satisfactorily [13, 16]. Another area for improvement in OL is the quality of student engagement in various educational activities compared to face-to-face learning. Learning engagement was positively connected with the intended academic outcomes, strong academic performance, student satisfaction, and perseverance [17, 18]. Student engagement is the foundation for understanding and knowledge formation. Students who actively participate form intellectual and emotional habits that prepare them for lifelong learning [19]. Administrative concerns, learners’ motivation, availability of time and resources for study, and cost are some factors to consider [20]. Low-quality OL can also be caused by ineffective multimedia material design and arrangement [21].

The significance of assessing a student’s readiness for OL before they join an online course is vital and has been supported by previous studies [22–25]. Technological competence and internet access are considered essential to the success of OL, along with other criteria like learning outcomes and learners’ satisfaction [25, 28]. The importance of social skills for students’ academic success has also been emphasized [29, 30]. It was observed that students’ effectiveness in OL was substantially connected with their ability to interact socially with their teachers and peers. Effective interpersonal and communication skills significantly impact academic success [31–33].

Many online medical education courses have yielded results on par with on-campus courses [34, 35]. The medical students accepted online classes, which offers a significant and encouraging possibility for the future of medical education [4, 36, 37, 38]. The COVID-19 crisis has provided educators with the opportunity to advance their understanding of OL platforms and digital media production, as well as to create unique assessment strategies and change educational principles [39–42]. During the pandemic, OL garnered positive student feedback [36, 43–45]. However, certain studies [46–49] have documented students’ negative OL experiences during the pandemic. For example, according to dental students at Harvard University, learning and engagement suffered during the pandemic [50, 51], while Pakistani medical and dentistry students noted fewer interactions between students and instructors [46].

Now that teaching is returning to the “new normal,“ we want to identify the areas under the implemented OL that are particularly beneficial for future use in specific pre-clinical courses. In order to raise the quality of the teaching-learning process, it is necessary to evaluate student’s learning performance and satisfaction in OL. This study considered how OL was delivered by the College of Medicine’s pre-clinical Foundation Course (FB) at Princess Nourah bint Abdulrahman University (PNU). PNU is a public women’s university and the world’s largest women’s university in Riyadh, the Saudi capital. Did OL meet the course learning objectives? Should OL be used in some pre-clinical courses after COVID? The results of this empirical investigation could provide a strong foundation for future curriculum improvement and implementation. The current study covered the following goals.

1. To assess the perceived readiness for OL among medical and dentistry students using SLOR.
2. To compare the quality of students’ course performance in online versus face-to-face learning as measured by their grades in theory and skills.
3. To evaluate students’ level of satisfaction with the quality of the implementation of online learning.
4. To determine whether students’ online learning readiness affects their academic achievement in theory, skills, and satisfaction.

## Methods

### Study design

This descriptive comparative study compared students’ academic performance in a face-to-face and online setting. The first semester grades of students who took classes online from September through November 2020 were compared to those who took face-to-face classes from September through November of the following academic year, 2021. Additionally, the Student Online Learning Readiness (SOLR) questionnaire [25] was used to assess students’ readiness for OL at the start of FB. At the conclusion of the course, a self-made survey questionnaire was used to evaluate how satisfied the students were with the online delivery method.

### The study participants

A purposive sampling technique was employed in which all first-year medical and dental students enrolled during the first semester of the academic year 2020–2021 participated in the study. One hundred twenty-one 121 students (78 medical and 43 dentistry) enrolled in the 10-credit Foundation Course (6 h of theory and 4 h of practical) from September to November 2020 participated in the study, except those students who dropped the course.

### Implementation of online and face-to-face learning

Students were taught in the face-to-face modality following a unique problem-based hybrid curriculum. The traditional lecture method consists of didactic PowerPoint presentations in classrooms. Problem-based learning (PBL) and self-directed learning (SDL) were also included. Practical and simulation sessions were conducted in different laboratories depending on the subjects (Anatomy, physiology, Biochemistry, Microbiology, Pathology, and Clinical Simulation Lab).

In the OL modality, students obtained theoretical and practical education entirely OL via the Microsoft Teams and Blackboard CollaborateTM platform. The Blackboard system provides a valuable educational environment where students can interact socially and academically with each other and staff [26]. Most lectures were delivered by faculty synchronously, and some through recorded videos. Practical and simulation sessions were conducted through online synchronous lab demo sessions and video presentations. Daily OL class was generally 6 h. The tutors regularly schedule PBL small group sessions to encourage social interaction in OL sessions. Interactions via the “Discussion Board” were done, like posting of open-ended-questions regarding a PBL case. There was also a scheduled “Online” consultation time via Microsoft Team Channel.

The same instructors taught Medical and dental students using similar references and content. Supporting materials like videos and Microsoft PowerPoint Presentations of the lectures were provided to the students in both modalities through the Blackboard CollaborateTM platform at least 24 h before each lecture was delivered as per the policy of the Basic Science Department of the College of Medicine. All instructors underwent a series of mandatory seminars, workshops, and training regarding teaching strategies and assessment methodologies to deliver the OL and face-to-face modalities.

### Assessment of Outcomes

The results obtained in quizzes, midterm, and final examinations were used to evaluate students’ academic performance in theory. The scores achieved in OSPE were used to evaluate the skills learned. OSPE was carried through the ExamSoft® platform using the same specimens in the dissection labs and models shown in the synchronous online practical session. Students were tested using the same evaluation methods and questions in online and face-to-face groups. Three filter standards are set by the Assessment Unit to be followed. The ***First filter*** is an intradepartmental assessment meeting based on specialization where all instructors in each department meet to evaluate the exam questions in terms of content and course learning outcomes achievement; ***Second filter*** is an interdepartmental assessment meeting with the subject expert to assess the content validity of the questions further; and the ***Third filter*** is the Exam Committee composed of the DBS Quality Coordinator, Assessment Unit Head, Course Chair and Co-chair for final evaluation and approval based on the prepared blueprint. All assessments were conducted via the ExamSoft® platform, a computer-based assessment software that simplifies the exam process, collects assessment data and generates reports to help faculty improve the course and student performance. It is used by more than 180 medical programs throughout the world [27].

### Data Collection

Data on students’ readiness for OL was gathered using a validated Students’ Online Learning Readiness Survey (SLOR) questionnaire [25]. Students were given a link to the online questionnaires, which can be completed independently. Before data collection, the study participants were informed about the research’s goals and how it would be carried out. Their voluntary participation in the study was emphasized, and they could withdraw anytime they wanted.

The SLOR questionnaire is composed of four components that measure students’ readiness for OL; technical competence, social competence with the instructor, social competence with classmates, and communication. Technical competency is measured by six items initially from other instruments [2], then adapted and modified to evaluate students’ technical competencies. Social competencies with the instructor in OL include five items, and social competencies with classmates include five items, both of which were from the previous instrument [52]. These items enhanced the distance learner’s sense of belonging in online courses and positively correlated with academic achievement [25]. Four items are included for measuring communication competencies in OL.

Technical competence was assessed by looking at how proficient students were in a wide range of computer technologies, their confidence in their ability to use them for specific tasks, and how comfortable they were with computers. It also considers students’ capacity to incorporate computers into their learning activities and whether or not they are motivated to participate more in learning activities while utilizing computers.

The social competence with the instructor was gauged by how confident the students were in social interaction with the instructors by respectfully initiating discussions and, asking them questions, seeking help when needed. Social competence with classmates was evaluated by how they initiate and interact with other students with respect and develop friendships with their classmates. Other factors considered were how students pay attention to other students’ social actions and how they apply different social interaction skills depending on the situation.

Communication competence was determined by how comfortable students expressed their opinion through writing and speaking to others. It includes how they respond to other people’s ideas, their ability to express an opinion in writing so that others understand, and their proficiency in giving constructive and proactive feedback to others even when they disagree.

Internal consistency was assessed in the current study using Cronbach’s alpha coefficient, which produced results ranging from 0.74 to 0.87, indicating an adequate level of internal consistency. A five-point Likert scale (1 = Disagree, 2 = Tend to disagree, 3 = Neutral, 4 = Tend to agree, 5 = Agree) was used to measure the competency level for each item. In the presentation of data, “disagree and tend to disagree”, with a Likert scale mean score of 1.0-2.4, were considered “Not Ready” for OL; 2.5–3.4 for “Neutral or Undecided”; and 3.5-5.0 representing being “Ready” for OL.

Students perceived satisfaction with the quality of OL implementation was assessed using a survey questionnaire prepared by the authors with reference to the Course Evaluation Questionnaire (CEQ). This questionnaire is used by the Department of Basic Sciences of the College of Medicine to evaluate the quality of each course at the end of its implementation. The CEQ is, however, designed to evaluate face-to-face learning modality; hence, we prepared a questionnaire that would appropriately assess the student’s level of satisfaction in OL. The prepared satisfaction questionnaire considered the following aspects of instruction: (1) Organization and management of the course; (2) Effective application of technology; (3) Interaction with instructors and course staff; (4) Interaction with classmates and groupmates; (5) Acquisition of knowledge; and (6) Acquisition of skills.

Two medical education experts, a PhD in education focused on curriculum and teaching, and a Doctor of Education (D. Ed.), validated the questionnaire. Interaction with classmates and group members, item number 4, was added to the questionnaire due to a validator’s recommendation.

The validated instrument was pilot tested with the participation of 15 medical and dental students. A few improvements, such as making the instructions explicit, were based on the feedback from the pilot study. The final results did not incorporate the data acquired during the pilot test. The self-made satisfaction questionnaire’s internal consistency was evaluated using the coefficient alpha or Cronbach’s alpha. The yielded Cronbach’s alpha coefficients range from 0.78 to 0.89, demonstrating an acceptable internal consistency.

A five-point Likert scale: 1 = Not satisfied, 2 = Partly satisfied, 3 = satisfiedl, 4 = more than satisfied, 5 = Very satisfied was used to evaluate students’ level of satisfaction with OL, which was conducted at the end of FB.

Comparative analysis of students’ academic performance online versus face-to-face was anchored on FB course assessments, including quizzes, midterm exams, final exams, and Objective Structures Practical Exams (OSPE). Quizzes include Multiple Choice Questions (MCQs) and True or False questions, while the midterm exam comprises MCQs, Short Answer Questions (SAQs), and the final exam includes MCQs. In both groups, the students received the same evaluation methods and questions.

### Data Analysis

Quantitative data analysis was conducted after obtaining all results of the online survey. Descriptive statistics in terms of means, frequency, percentage and standard deviations, t-test, and multiple regression analysis were used to describe and analyze the data gathered. SPSS version 26 was used for data entry and analysis with a P-value ≤ 0.05 was considered statistically significant.

## Results

### Students perceived readiness for online learning

**Fig. 1:**
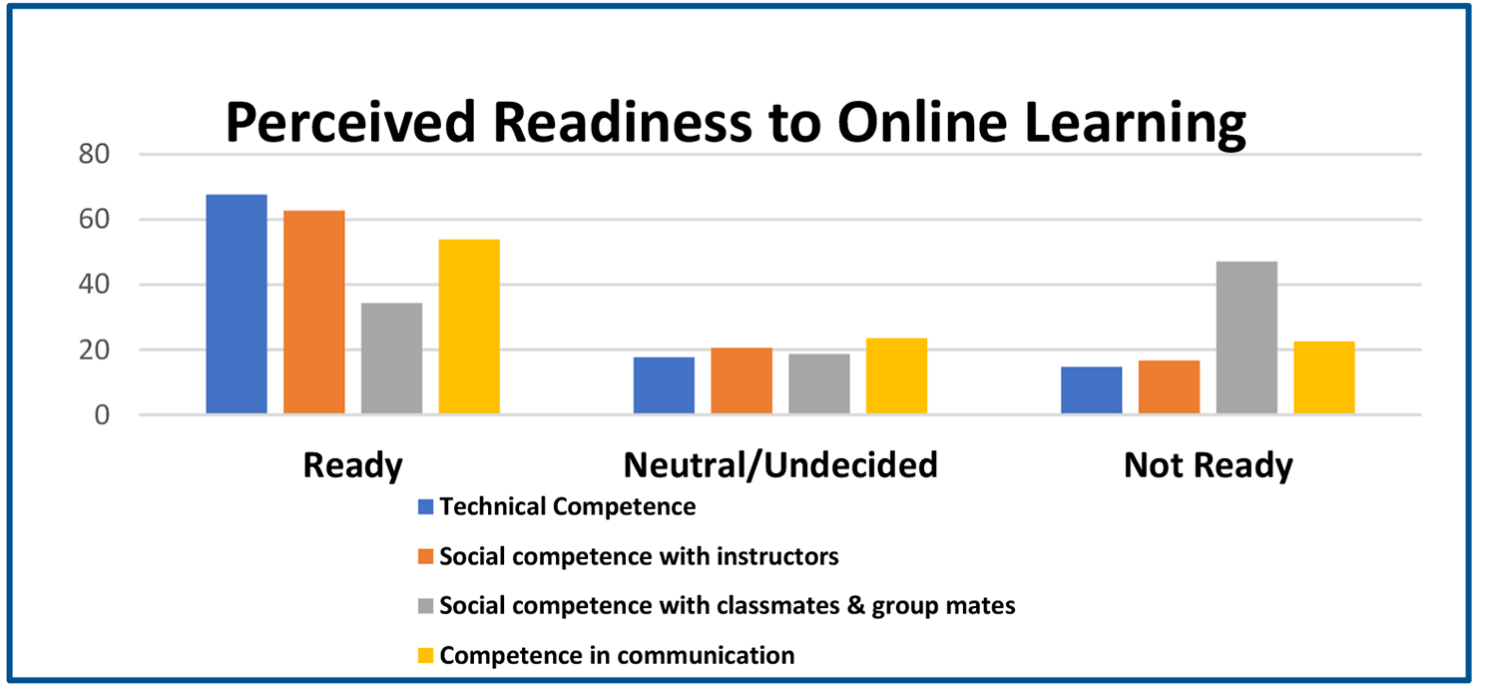
Frequency distribution of perceived competence/readiness to online learning

Findings in Fig. 1 show that more than 50% of medical and dentistry students are ready for OL, except for social competence with classmates and group members, where 47.05% of respondents needed to prepare. Technical aptitude was highly rated among the students.

### Comparison of students’ academic performance in online and face-to-face modalities

Students’ face-to-face academic performance in the previous academic year is comparable to that of the students in the current study. For the preceding academic year, students’ mean score for 40% theory/continuous assessment was 36.23; for 40% theory/exams, they scored 34.71; and 18.02 for 20% skills/OSPE. It is important to note that medical and dental students enrolled in the FB course were in the top 20 to 30% of the preparatory year program of PNU in terms of academic achievement.

Table 1 shows no significant difference in students’ academic performance in OL and face-to-face learning when analyzed according to the exam (p = 0.152) and continuous assessments (p = 0.163). However, there is a significant difference in the skills acquisition of students who learned face-to-face compared with OL, with the former receiving significantly (p = 0.029) higher scores. These results indicate that students acquire skills more effectively through face-to-face learning than OL.

**Table 1:** Test of difference of students’ academic performance in online and face-to-face modalit**ies**

Assessment Methods	Modality		Mean	t-value	p-value	Interpretation
Theory/Continuous Assessment/ (40%)		Online	36.83	1.773	0.152	Not Significant
		Face-to-face	36.56			
Theory/Exams (40%)		Online	34.83	1.660	0.163	Not Significant
		Face-to-face	34.46			
Skills/OSPE (20%)		Online	16.29	4.453	0.029**	Significant
		Face-to-face	17.87			

**Significant at p ≤ 0.05

### Level of satisfaction with OL implementation

**Fig. 2:**
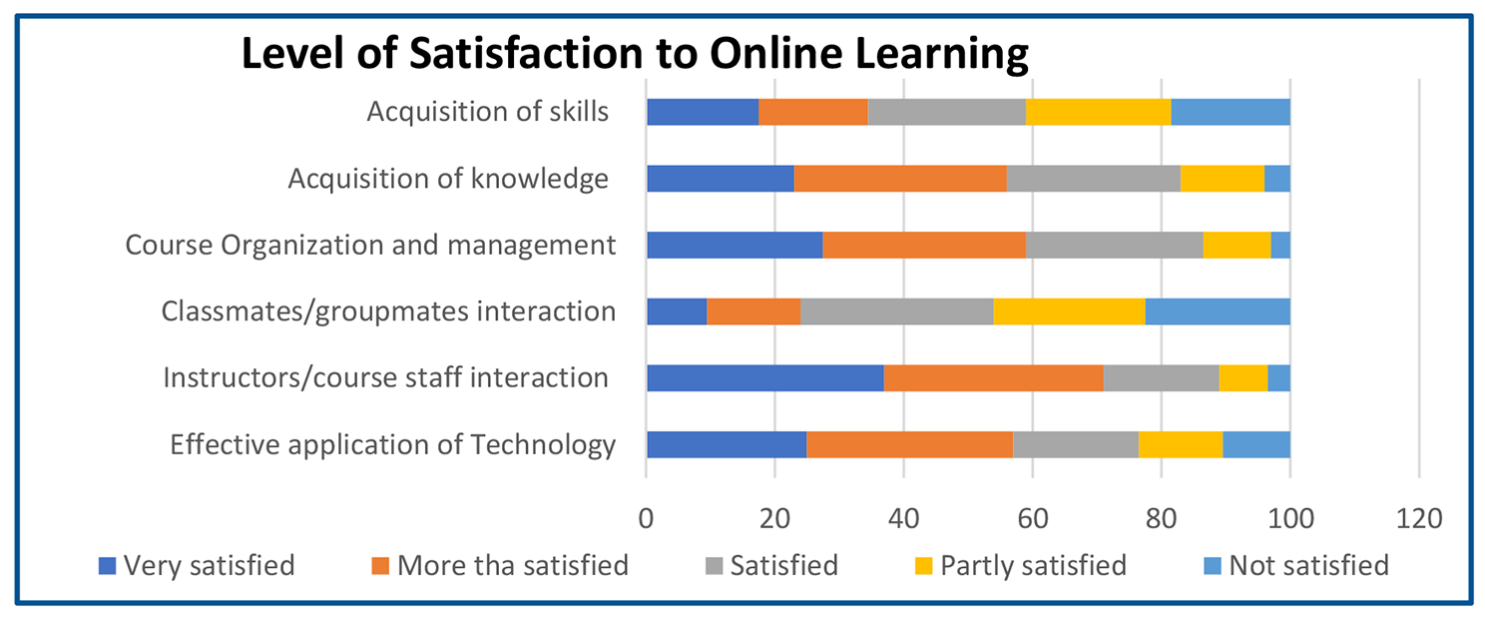
Frequency distribution on the level of satisfaction with online learning implementation

As reflected in Fig. 2, students gave the highest satisfaction rating in OL for interaction with faculty and staff, followed by course organization and management, effective application of technology, and acquisition of knowledge. Students were less satisfied with the acquisition of skills. The lowest satisfaction scores were obtained from interaction with classmates and groupmates and skills acquisition.

### Effects of perceived online readiness on students’ academic performance and satisfaction in online learning

The multiple regression analysis results in Table 2 revealed that technical and communication competence are not statistically significant predictors of academic performance (p > 0.05). However, the results showed a statistically significant association between social competence with instructors (p = 0.021) and social competence with classmates (p = 0.007) with students’ academic performance. The multiple regression results reflect that social competence with instructors and classmates envisaged students’ academic performance.

**Table 2:** Multiple regression results between academic performance and their perceived online readiness

SOLR Components		Coefficients	Std. Error		t-value	p-value
{Constant) Technical Competence Social Competence with Instructors Social Competence with Classmates Communication Competence		14.250 0.207 0.889 -1.092 0.421	1.673 0.359 0.380 0.396 0.453		8.515 0.577 2.342 -2.760 0.929	0.000 0.565 0.021** 0.007** 0.355
	df	Sum of Squares	R^2^	R^2^(adj)	F	Sig
Regression Residual Total	4 97 101	64.525 611.251 675.776	0.095	0.058	2.560	0.043**

**Significant at p ≤ 0.05

Table 3 shows the results of the multiple regression analysis, which revealed that technical, communication, and social competence with classmates are not statistically significant predictors of academic performance (p > 0.05). But the results showed a statistically significant association between social competence with instructors (p = 0.003) with students’ academic performance. Results of the multiple regression show that interaction with instructors significantly (*p = 0.003*) predicts students’ satisfaction with OL.

**Table 3:** Multiple regression results between the perceived online readiness and students’ satisfaction with OL

SOLR Components		Coefficients	Std. Error		t-value	p-value
{Constant) Technical Competence Social Competence with Instructors Social Competence with Classmates Communication Competence		2.911 -0.090 0.210 0.079 0.082	0.307 0.066 0.070 0.073 0.083		9.469 -1.364 3.012 1.092 0.984	0.000 0.176 0.003** 0.278 0.328
	df	Sum of Squares	R^2^	R^2^(adj)	F	Sig
Regression Residual Total	4 97 101	8.784 20.635 29.419	0.299	0.270	10.322	0.000**

**Significant at p ≤ 0.05

## Discussion

This study examined how prepared medical and dentistry students were for OL, how they performed academically, and how satisfied they were with it. Prior studies have emphasized how crucial it is to gauge students’ readiness for OL before they enroll in a course [22, 24, 25]. It was said that students’ preparedness for OL significantly impacted their academic success [53, 54].

Above 50% of students demonstrated readiness for OL except in social competence with classmates and groupmates, with more than 50% scoring low in this dimension. These results imply that many students need improvement in interacting with their classmates. This result corroborates previous research findings that the OL modality may lead to social isolation because it does not provide enough opportunities to interact with other students [36]. The lack of opportunities for students to interact with others and hone their interpersonal skills has been identified as one of the disadvantages of OL in earlier studies. Some people expressed concern that online learners are more likely to feel lost, alone, and frustrated, which could hinder or lessen their ability to learn effectively. Discussion and interaction between students and teachers are also limited [13–16]. When social interaction is lacking, students are more likely to drop out or become disinterested in their courses [55].

In light of the critical role of social interaction in OL, faculty are urged to employ interactive features of OL platforms to provide clear channels for student-instructor and student-student interactions [37]. Students’ relationships with classmates matter for their learning, especially when face-to-face social interactions are limited. Therefore, students’ social competence with classmates must be developed in OL. The instructors must consider the diversity in students’ social needs, especially those who appear passive or absent during synchronous lectures and other academic activities. OL activities, tutorial support, and on-time feedback on students’ academic performance may also be intensified to enhance social interaction. Moreover, technological competence, like being comfortable and confident in using various computer technologies, is also considered a significant factor for the achievement of OL, including learning outcomes and learner satisfaction [28, 56].

To enhance students’ social interaction and address their difficulty in skills acquisition, they were divided into small PBL groups to study and interact with each other online. This was done by setting up channels on the Microsoft Teams platform where they could engage, communicate, and collaborate in those teams to resolve some PBL problems, create assignments, and revise together. Moreover, the skills acquisition gaps were identified when face-to-face instruction was resumed, and all crucial concepts and clinical skill sessions were made up for in other blocks by adding extra practical and tutorial sessions.

There was no significant difference in students’ academic performance in theory in OL and face-to-face learning. However, face-to-face learning was considerably better than OL in terms of practical skills as measured by OSPE (p = 0.029). In a prior study [57], the academic performance of the retrospective control group in the first semester of 2019 was compared to a prospective experimental group in the first semester of 2020. The results indicated no significant difference between the knowledge and ability levels of students taught using traditional or OL approaches. However, students in the OL group showed somewhat higher knowledge and competence ratings than traditional, face-to-face group students. In a survey of dental students, those who took OL courses performed better on average than those who took traditional learning courses [58].

The results of the current study differ from what has been reported in several published articles [59, 60], which provide evidence that OL is feasible and effective in learning basic practical skills in novices. Our students in FB obtained significantly higher practical skills grades face-to-face than those who took the OL. Although OL is supported by technological proficiency, other elements also play a role in the success of the delivery of practical skills online. Teaching practical skills requires the instructor’s physical presence to watch students closely and correct their errors until they reach the appropriate level of competency [61]. One way to accomplish this is to use effective feedback techniques and monitoring tools, such as teleconferencing, that enable real-time communication between the students and instructors during OL practical skills sessions [62]. In our case, more than the time and opportunity provided for online interactions between students and instructors during practical sessions may be required to ensure the necessary level of competency. Additionally, our students were not attuned to practical OL implementation, which may have hindered their skills’ learning efficiency. Our practical skills have always been delivered through hands-on experience and procedural simulation sessions. Hence, OL of practical skills is still a great challenge for us should we adopt the OL modality.

It is essential to draw attention to various published works that discussed how some students were skeptical that OL was an appropriate method for developing practical competence. For example, it was reported in one study that most dental and medical students agreed that online courses were less successful than in-person courses [63]. Even more, emphasis was placed on the fact that while the OL is a suitable delivery method for the theoretical components of medical curricula, the practical aspects must be carried out in person to promote the development of psychomotor abilities [64].

Students generally expressed the highest satisfaction score with OL concerning “interaction with course instructors and staff”. More than Lower satisfaction scores were obtained from “interaction with classmates and groupmates”. Since we are from a female university, all of the participants in our study are female, which may account for their high level of satisfaction with student-faculty interaction. Studies have shown that female students are more engaged in OL and have a greater level of self-regulation and the capacity to deal with challenges associated with OL [65]. Female students are more likely to use various online tools to interact with teachers and ask for support throughout online education [66].

The current study’s findings showed that students expressed higher satisfaction with teachers and staff may be explained by various reasons. The faculty made extra efforts by incorporating more interactive tools into the teaching process to monitor students’ learning needs and queries. In Microsoft Teams and Black Board, several channels have been created so students can quickly contact the teachers and receive a prompt response. Hence, students can address their concerns related to academic activities required in the course. Instructors were fully aware of the potential consequences of decreased interaction between them and students during OL; hence extra effort was made to address this potential deficiency. Additionally, “informal” methods of communication through Microsoft Teams, Black Board, and even “WhatsApp” may have helped students meet their expectations for involvement with their course instructors. The unfulfillment of the students’ desired interaction with their classmates, similar to during face-to-face interaction, may have resulted in lower student satisfaction scores in “interaction with classmates.“ This suggests that students need more time to be ready for social involvement with their classmates. Due to the hybrid PBL curriculum used in the FB, students are separated into smaller groups of 10 to 12 for PBL case discussions. We anticipated that the small group sessions would provide a venue to improve social interaction, but our findings do not support this. It is possible that their commitment to this smaller group was insufficient to improve their ability to interact socially with other students.

The results of the current study indicated that social readiness with instructors and classmates envisages students’ academic performance. Likewise, interaction with instructors significantly predicts students’ satisfaction with OL. These results imply that social interaction in the academic context is relevant to students’ learning. Another study indicated that a lack of student engagement suggests poorer academic achievement [67]. These results also corroborate the findings in another study where there is a positive correlation between students’ perceived engagement with faculty and classmates and the overall effectiveness of the online course [37].

The results of this study demonstrate that students perform substantially better academically in theory in OL but not in practical skills. Instructional interventions should be applied to address gaps, like promoting academic and social interaction through varied educational strategies to foster social presence and engagement. In medical education, we deemed it necessary to teach practical and clinical skills in the face-to-face modality. It is vital to use instructional interventions to overcome barriers to OL, such as fostering academic and social interaction using various pedagogical techniques that encourage social presence and participation.

### Limitations

There were limitations regarding this study, one of which is in regard to the sample, which is only focused on one university for women. Thus, the sample is gender imbalanced. It is therefore recommended to conduct a study with students from different universities, including male respondents, to address statistical sampling bias. Another limitation pertains to the SOLR instrument used to explore students’ readiness or competence to OL. This instrument was developed to assess students’ perceived competencies, not observed competencies, which can be subjective. Hence, evaluating students’ perceived competencies and comparing them with their effective performance is suggested. To address this possible bias, the present study investigated students’ academic performance in theory and skills vis-à-vis their online learning readiness or competency.

## Conclusion

Despite the difficulties in delivering OL, there is a remarkable chance of providing high-quality online instruction for theoretical components of medical curricula, even in the post-covid period. However, the practical and clinical components must be executed through a face-to-face modality. When implementing OL, academic leaders and curriculum developers must be aware of potential gaps unique to each university or institution. The faculty development program that will support and facilitate the online course design implementation must be considered. Additionally, it is crucial to continuously evaluate the various domains concerning the delivery of OL to address any implementation-related challenges quickly. To enable the deployment of OL, institutional policies regarding it must be in place and be given clear direction.

## Data Availability

The data used in this study are available and will be provided by the corresponding author on a reasonable request.
